# Effects of HOXC8 on the Proliferation and Differentiation of Porcine Preadipocytes

**DOI:** 10.3390/ani13162615

**Published:** 2023-08-14

**Authors:** Weiguo Cui, Qian Zhang, Hanqiong Wang, Xiaohan Zhang, Ming Tian, Di Liu, Xiuqin Yang

**Affiliations:** 1 College of Animal Science and Veterinary Medicine, Heilongjiang Bayi Agricultural University, Daqing 166319, China; 2 College of Animal Science and Technology, Northeast Agricultural University, Harbin 150030, China; 3 Institute of Animal Husbandry, Heilongjiang Academy of Agricultural Sciences, Harbin 150086, China

**Keywords:** HOXC8, preadipocyte, adipogenesis, proliferation, RNA-seq

## Abstract

**Simple Summary:**

Fat accumulation is important for both livestock production and human health. Previous studies provide a clue to the involvement of Homeobox C8 (HOXC8) in adipogenesis. Here, we make clear that porcine HOXC8 promotes the proliferation and differentiation of preadipocytes through gain- and loss-of-function approaches. Furthermore, the genes and pathways affected by HOXC8 during the regulation of preadipocyte proliferation and differentiation are identified. The results provide new data for the clarification of mechanisms underlying fat accumulation, which is the basis for controlling fat contents in mammals.

**Abstract:**

Transcription factor Homeobox C8 (HOXC8) is identified as a white adipose gene as revealed by expression profile analysis in fat tissues. However, the specific role of HOXC8 in fat accumulation remains to be identified. This study was designed to reveal the effects of HOXC8 on preadipocyte proliferation and differentiation. We first make clear that the expression of HOXC8 is associated with fat contents in muscles, highlighting a role of HOXC8 in fat accumulation. Next, it is demonstrated that HOXC8 promotes the proliferation and differentiation of preadipocytes through gain- and loss-of-function assays in primary cultured porcine preadipocytes. Then, mechanisms underlying the regulation of HOXC8 on preadipocyte proliferation and differentiation are identified with RNA sequencing, and a number of differentially expressed genes (DEGs) in response to HOXC8 knockdown are identified. The top GO (Gene Ontology) terms enriched by DEGs involved in proliferation and differentiation, respectively, are identical. IL-17 signaling pathway is the common one significantly enriched by DEGs involved in preadipocyte proliferation and differentiation, respectively, indicating its importance in mediating fat accumulation regulated by HOXC8. Additionally, we find that the inhibition of proliferation is one of the main processes during preadipocyte differentiation. The results will contribue to further revealing the mechanisms underlying fat accumulation regulated by HOXC8.

## 1. Introduction

Fat is one of the most important traits for farm animal production. Excessive deposits of fat are negatively associated with feed remuneration, growth rate, carcass quality, and lean meat percentage [1], leading to a decrease in economic benefits. However, intramuscular fat (IMF) contributes to the flavor, tenderness, and juiciness of meat, and is favored by consumers [2]. A higher amount of IMF and a lower total fat content, therefore, are of benefit to livestock. Controlling fat content and distribution has become one of the major goals for livestock producers and breeders, for which understanding the mechanisms underlying fat deposition is essential. Additionally, the increasing prevalence of obesity and related metabolic diseases in the human population also makes it urgent to reveal the mechanisms underlying fat deposition.

Adipose tissue is the major player in fat deposition. The expansion of adipose tissue depends on two events: the increase in the number of new adipocytes (hyperplasia) and size of existing adipocytes (hypertrophy) caused by the increased accumulation of lipids [3]. Hyperplasia results from the proliferation and/or differentiation of stem cells/preadipocytes. Intensive efforts have been made to reveal the genetic basis of hyperplasia, especially in terms of differentiation, and several transcription factors (TFs) including the master adipogenic markers, peroxisome proliferator-activated receptor γ (PPARγ) and CCAAT/enhancer-binding protein α (C/EBPα) [4,5], have been characterized as regulators of adipogenesis. However, adipogenesis is a tightly orchestrated cellular differentiation process regulated by a cascade of TFs; the regulatory network of adipogenesis is far from complete.

Homeobox (HOX) gene family encode homeodomain-containing TFs are evolutionarily conserved and play a critical role in maintaining cellular identity and regulating differentiation [6,7,8]. The HOX members were not found to be associated with adipogenesis until 1997, when researchers found that three members of the family, HOXA4, HOXA7, and HOXD4, were differentially expressed in adipocytes [9]. Now, studies have revealed that some members of the HOX family such as HOXC10 and HOXB7 may be associated with adipogenesis through functional analysis and/or expression profile characterization [10,11,12,13].

As for HOXC8, it was categorized as a white adipose gene. It is expressed more highly in white adipose tissue (WAT) than in brown adipose tissue (BAT) [14,15]. It is the most highly expressed among 40 members of the family, as revealed by RNA sequencing (RNA-seq) in human fat progenitor cells obtained from flank subcutaneous adipose tissue [16]. Additionally, the expression of HOXC8 is depot-dependent in WAT, that is, differentially expressed in different depots such as subcutaneous and visceral preadipocytes [17]. These results indicate the expression of HOXC8 is highly regulated in adipose tissue and it might be important for adipogenesis. Factually, HOXC8 was characterized as a gatekeeper of brown adipogenesis because miR-196a induces brown adipogenesis in WAT through the suppression of HOXC8 in vivo in mice [16].

However, little is known on the specific role of HOXC8 in white adipose accumulation, and the underlying mechanisms remain to be clarified. Here, we show that HOXC8 promotes the proliferation and differentiation of primary preadipocytes cultured from newborn Min pigs. Furthermore, RNA-seq is used to reveal the downstream genes regulated by HOXC8 during preadipocyte proliferation and differentiation. The results will contribute to further revealing the mechanisms underlying preadipocyte proliferation and differentiation regulated by HOXC8.

## 2. Materials and Methods

### 2.1. Animals, Samples, and cDNA Synthesis

Min pigs, an indigenous pig breed in China, were used and provided by the Institute of Animal Husbandry, Heilongjiang Academy of Agricultural Sciences (Harbin, China). For gene expression analysis, the samples including the heart, liver, spleen, lung, kidney, fat, muscle, intestine, and stomach were collected from 6-day-old pigs immediately after slaughter. Fat tissues were also collected from pigs at 30 and 210 days old. At each time period, three individuals were used. The samples were snap-frozen in liquid nitrogen and then stored at −80 °C. For the isolation of preadipocytes, the fat tissues were collected from newborn piglets and treated freshly. All animal treatments were approved by the Animal Care Committee of Northeast Agricultural University (Harbin, China).

Total RNA was isolated from tissues or cells with Trizol reagent (Invitrogen, Carlsbad, CA, USA) according to the manufacturer’s protocol. Reverse transcription (RT) was performed with 100 ng total RNA template and Oligo (dT)20VN primer using the HiScript III 1st Strand cDNA Synthesis Kit (+gDNA wiper) (Vazyme, Nanjing, China) to synthesize first-strand cDNA.

### 2.2. Cell Culture

Preadipocytes were isolated from newborn Min pigs as described [18] previously. Briefly, subcutaneous fat tissues were cleaned, cut into small pieces, and digested with 0.1% type I collagenase (Invitrogen) at 37 °C. The cells were then mixed with medium containing 1% penicillin–streptomycin (Invitrogen) and 10% fetal bovine serum (Sigma, St. Louis, MO, USA), and filtered through 400-mesh filters. The cells were cultured in Dulbecco’s modified Eagle’s medium/Nutrient Mixture F-12 (DMEM/F12) containing 10% FBS and 1% penicillin–streptomycin. The medium was changed every 2 days.

### 2.3. Preadipocyte Differentiation and Oil Red O Staining

When they reached 80–90% confluence, the preadipocytes were induced into differentiation with DMEM/F12 medium containing 10% FBS, 0.5 mmol/L 3-isobutyl-1-methylxanthine, 1 μmol/L dexamethasone, and 5 μg/mL insulin for 2 days. Then, the cells were transferred into maintaining medium composed of DMEM/F12 supplemented with 10% FBS and 5 μg/mL insulin for further culture. At 8 days postinduction, the mature adipocytes were stained using an Oil Red O kit (Leagene, Beijing, China), and morphologically viewed under a light microscope (Carl Zeiss AG, Jena, Germany). To quantify the lipid droplets, cellular Oil Red O was extracted using isopropanol and analyzed with optical absorbance at 510 nm.

### 2.4. Expression Plasmid, siRNA, and Transfection

The complete coding sequence of porcine HOXC8 was amplified with cDNA obtained from fat tissue. PCR was performed in a final volume of 20 μL containing 1 U rTaq DNA polymerase (Takara, Dalian, China), 1 × PCR buffer, 0.2 μmol/L of each primer, 200 μmol/L of each dNTP (Takara), and 1 μL of cDNA. The thermal cycling conditions were as follows: 94 °C for 5 min, followed by 30 cycles at 94 °C for 30 s, 56 °C for 30 s, 72 °C for 1 min, and a final extension at 72 °C for 10 min. The products were inserted into pCMV-HA at enzyme sites of *Eco*R I and *Kpn* I. The plasmids were verified by sequencing in Beijing Genomics Institute (BGI; Beijing, China).

The short interfering RNA (siRNA) against HOXC8 and nontargeting negative control (NC) were designed and synthesized by General Biosystems (Anhui, China). The optimal siRNA sequence was selected with real-time quantitative PCR (qPCR). The information for primers and siRNA is provided in Table S1. The plasmid/siRNA was transiently transfected into preadipocytes with Lipofectamine 2000 (Invitrogen) according to the manufacturer’s instructions.

### 2.5. Real-Time Quantitative PCR

qPCR experiments were performed with ChamQ Universal SYBR qPCR Master Mix (Vazyme) according to the manufacturer’s instructions, each in triplicate. β-actin was used as a reference. The amplification efficiencies were kept consistent between target and reference genes through primer designing. The relative expression level of target genes was calculated with the 2^−∆∆Ct^ method [19]. The primers were self-designed with primer premier 5.0 and synthesized in BGI (China). The primer sequences are listed in Table S2.

### 2.6. Western Blotting

Western blotting was performed as described previously [20]. Briefly, at 48 h post-transfection, the total protein was extracted from cells with RIPA (Beyotime, Shanghai, China) containing protease inhibitor (Invitrogen). A total of 25 µg of proteins was loaded on 8% SDS-PAGE for electrophoresis. The products were then transferred to a PVDF membrane (Millipore, MA, USA). Antibodies against HA tag (Catalog No. 66006-2-Ig) were obtained from Proteintech (Wuhan, China), and antibodies against β-tubulin (Catalog No. M30109M) were provided by ABMART (Shanghai, China).

### 2.7. Cell Counting Kit-8 Assay

The Cell Counting Kit-8 (CCK-8) assay was used to measure the proliferation of cells. Porcine preadipocytes were seeded in 96-well plates at a density of 5000 per well. When they reached 50% confluence, the cells were transiently transfected with plasmids overexpressing *HOXC8* or siRNA. At 24 h post-transfection, the cells were incubated with 10% CCK-8 (Beyotime) for 2.5 h at 37 °C. The optical density (OD) was measured at 450 nm with a Tecan Microplate Reader Infinite F50 (Tean GENios, Mannendorf, Switzerland). After being cultured for periods of time as indicated in the Results section, the cells were collected for CCK-8 analysis.

### 2.8. RNA Sequencing and Data Processing

siRNA against *HOXC8* and NC sequences was transiently transfected into preadipocytes, respectively, as described above. At 24 h after transfection, the cells were induced to differentiation. At 0 and 3.5 d postinduction, the cells were collected for the construction of paired-end RNA-seq libraries. At each time point, six libraries were constructed from treatment and NC groups, each with three. A total of 12 libraries were constructed. The RNA was isolated with Trizol reagent (Invitrogen) according to the manufacturer’s instructions. A NanoPhotometer spectrophotometer (IMPLEN, Calabasas, CA, USA) and an Agilent 2011 Bioanalyzer (Agilent, CA, USA) were used to assess the RNA quality. Library preparation and RNA sequencing (RNA-seq) were performed by the Frasergen Co., Ltd. (Wuhan, China) on a NovaSeq6000 sequence analyzer (Illumina, San Diego, CA, USA) according to the manufacturer’s instructions.

The RNA-seq data were processed as described previously [21]. Briefly, the raw reads were cleaned using SOAPnuke software (v2.1.0) [22] with default parameters. The clean reads were mapped to the pig reference genome (Sscrofa 11.1, http://asia.ensembl.org/Sus_scrofa/Info/Index, accessed on 6 March 2022) with HISAT2 (v2.1.0) [23] and the coverage of RNA-seq reads was calculated using the geneBody_coverage.py script of the RSeQC software (v2.6.4) [24]. The mapped reads were then assembled with StringTie software (v2.1.7) [25], and the resultant transcripts were quantified in fragments per kilobase million (FPKM) with Cufflinks (v2.2.1) [26]. The expression threshold was set at FPKM > 0.1 in at least one sample. The differentially expressed genes (DEGs) were identified with DESeq2 (v1.22.2) [23] and the threshold was set as absolute Log2 fold change (FC) ≥ 1, *p* < 0.05 (unless otherwise indicated). Gene Ontology (GO) and Kyoto Encyclopedia of Genes and Genomes (KEGG) enrichment analyses were performed to analyze the function of DEGs as described previously [21]. Search Tool for the Retrieval of Interacting Genes/Proteins (STRING) (https://cn.string-db.org/, accessed on 1 June 2022) was used to reveal the interaction between DEGs with default parameters, and the results were visualized with Cytoscape.

### 2.9. Statistical Analysis

All experiments were conducted at least three independent times, each in triplicate. Data were given as mean ± standard deviation (SD). SPSS19.0 (SPSS; Chicago, IL, USA) was used to analyze the data. The differences between groups were compared with Student’s *t*-test, while those among multiple groups were analyzed with one-way ANOVA followed by Ducan’s multiple comparison test. * indicates a significant difference (*p* < 0.05), and ** indicates a very significant difference (*p* < 0.01).

## 3. Results

### 3.1. Expression Profile of HOXC8 in Fat Tissues and Cells

**Figure 1:**
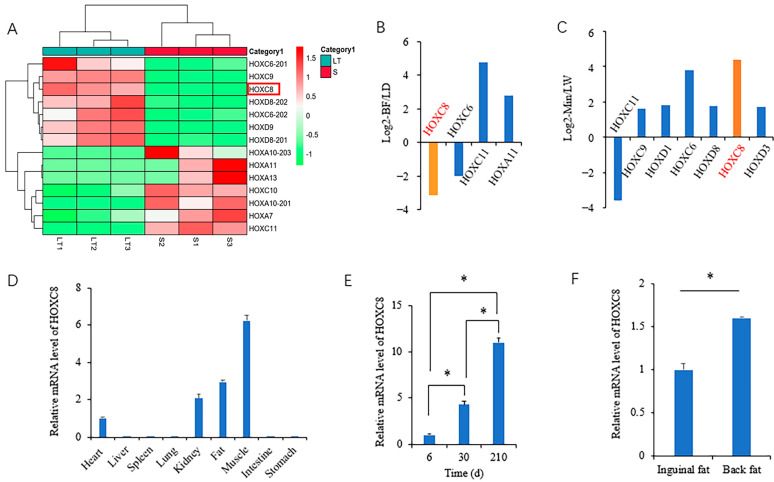
Expression profile of *HOXC8* in tissues. (**A**) Heatmap of HOX family members in *longissimus thoracis* (LT) and *semitendinosus* (S) muscles; (**B**) differentially expressed genes (DEGs) of HOX family members in *biceps femoris* (BF) compared to *longissimus dorsi* (LD) muscles (FDR < 0.01); (**C**) DEGs of HOX family members in *longissimus dorsi* muscles from Min pigs compared to those from Large White (LW) (FDR < 0.01); (**D**) expression profile of *HOXC8* in tissues; (**E**) developmental expression of HOXC8 in back fat of Min pigs; (**F**) expression of *HOXC8* in different fat depots of Min pigs. * *p* < 0.05.

We performed RNA-seq to identify fat-associated genes using muscles from different depots and pig breeds that differed in fat contents previously [21,27]. Through rechecking these data, we found that the expression of *HOXC8* was associated with the fat contents of muscle. Between muscles from Min pigs with differential IMF contents, *HOXC8* mRNA is more abundant in *Longissimus thoracis* (LT) than in *Semitendinosus* muscle (Figure 1A) and in *Longissimus dorsi* (LD) than in *Biceps femoris* muscle (Figure 1B) as well. In these two groups of comparison, both LT and LD have higher IMF contents than their respective control muscle. Between Large White and Min pigs, representatives of lean- and fat-type pig breeds, *HOXC8* showed a higher expression in LD from Min than that from Large White pigs (Figure 1C). Additionally, among all members of the family, *HOXC8* was the only one whose expression alteration was consistent with that of the fat contents in all the three paired samples. These suggest that HOXC8 should have a role in the fat accumulation of pigs.

To investigate the role of HOXC8 in fat deposition, we characterized the expression profile in tissues. The mRNA level of *HOXC8* was high in fat, muscle, and kidney tissues, with the most in muscle, lowest in the heart, and none detected in the other visceral tissues analyzed including the liver, spleen, lung, intestine, and stomach (Figure 1D). In fat tissues, the expression of *HOXC8* gradually increased with increasing age (Figure 1E), and the level was significantly different (*p* < 0.05) between inguinal and back fat tissues from pigs at 210 d old (Figure 1F), that is, differentially expressed between the two fat depots.

### 3.2. Effects of HOXC8 on the Proliferation and Differentiation of Preadipocytes

**Figure 2:**
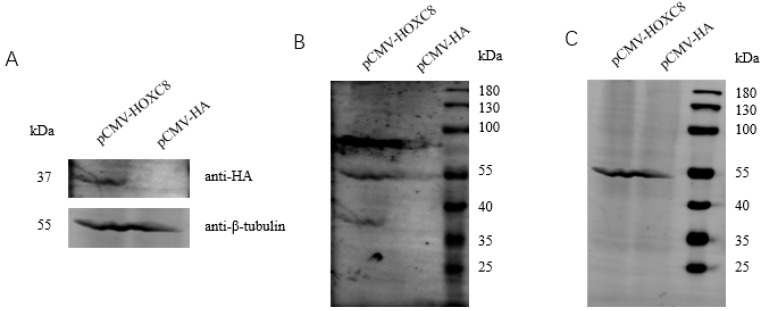

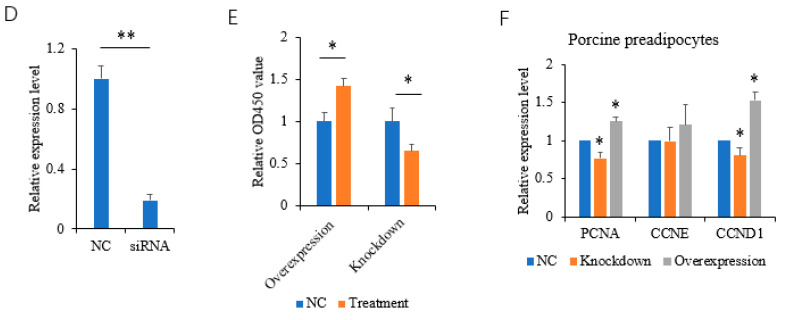
HOXC8 increases the proliferation of preadipocytes. (**A**) Efficiencies of vectors overexpressing HOXC8 as revealed by Western blotting in porcine preadipocytes; B and C, uncropped Western blots of anti-HA (**B**) and anti-β-tubulin (**C**) are shown in (**A**); (**D**) efficiencies of siRNA against HOXC8 detected with real-time analysis in porcine preadipocytes; (**E**) HOXC8 increases the proliferation of porcine preadipocytes as revealed by CCK8 at 48 h post-transfection; (**F**) effects of HOXC8 on the expression of genes associated with cell proliferation. * Significant vs. NC (*p* < 0.05); ** very significant vs. NC (*p* < 0.01).

The CCK-8 assay was used to analyze the proliferative effects of HOXC8 on porcine preadipocytes. We first analyzed the efficiencies of the overexpression vector and the synthesized siRNA (Figure 2A–C). siRNA-1824 can decrease the expression of *HOXC8* effectively and was therefore selected for the following experiments (Figure 2D). Overexpressing *HOXC8* increased, and knocking down *HOXC8* decreased, the proliferation of porcine preadipocytes significantly (*p* < 0.05) (Figure 2E). 

The expression of proliferation marker gene proliferating cell nuclear antigen (PCNA) [28], and the cell cycle genes cell cyclin D1 (CCND1) and CCNE [29] was analyzed. The *PCNA* and *CCND1* genes were positively regulated by *HOXC8* in porcine preadipocytes as revealed by the overexpression and knockdown experiments (*p* < 0.05). *CCNE* was not affected by HOXC8 in porcine preadipocytes (*p* > 0.05) (Figure 2F).

**Figure 3:**
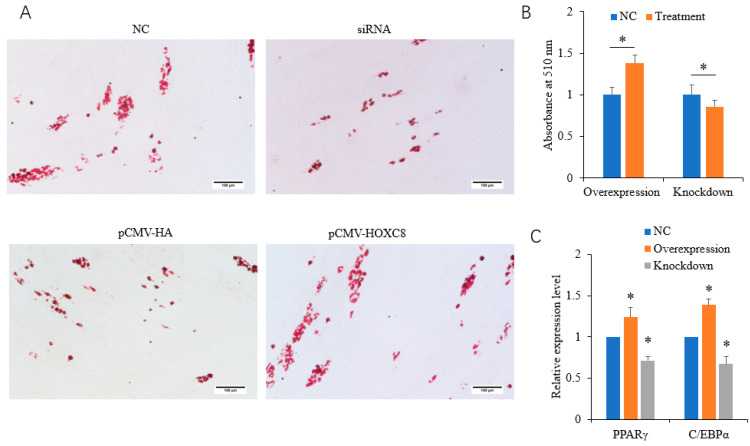
HOXC8 promotes the differentiation of porcine preadipocytes. (**A**) Results of Oil Red O staining at 8 days postinduction; (**B**) quantification of lipid contents at 8 days postinduction; (**C**) mRNA levels of adipogenic marker at 2 days postinduction. * Significant vs. NC (*p* < 0.05). The bar is 100 μm.

The overexpression of *HOXC8* promoted, and the knockdown of *HOXC8* inhibited, the differentiation of porcine preadipocytes, as revealed by Oil Red O staining and the quantification of lipid contents (Figure 3A,B). Consistently, the expression of the adipogenic markers C/EBPα and PPARγ [4,5] was increased in cells overexpressing *HOXC8* (*p* < 0.05) and decreased in cells interfering with *HOXC8* (*p* < 0.05) (Figure 3C).

### 3.3. Mechanisms Underlying the Regulation of Proliferation and Differentiation of Porcine Preadipocytes by HOXC8

**Figure 4:**
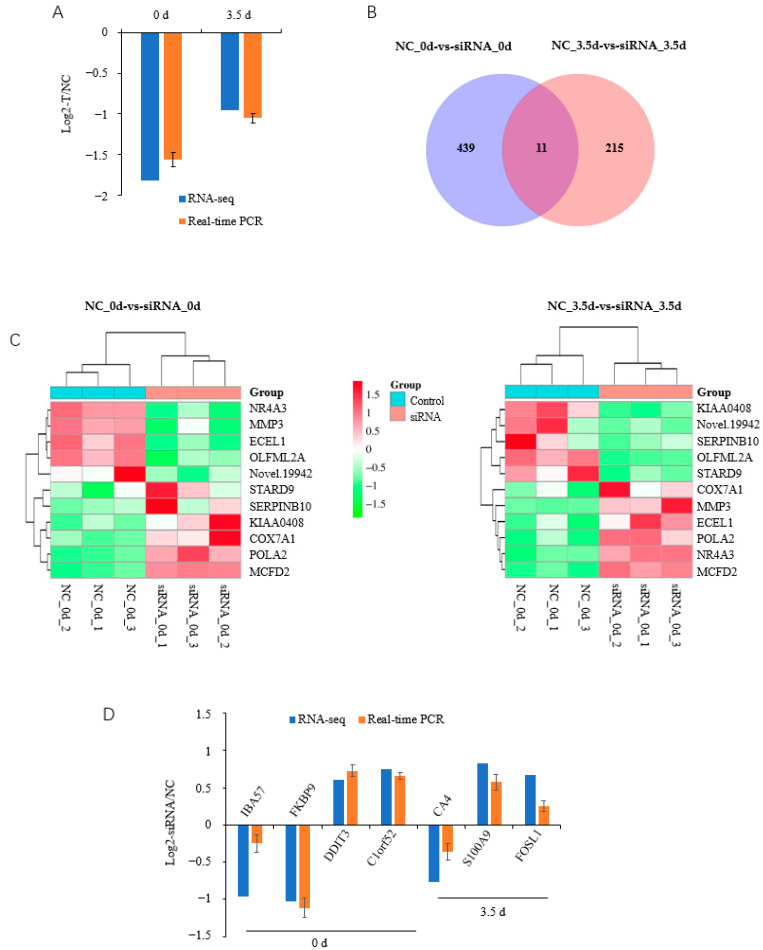
Overview of RNA-seq data. (**A**) *HOXC8* is effectively downregulated by siRNA during the differentiation of preadipocytes; (**B**) Venn diagram of differentially expressed genes in preadipocytes at 0 and 3.5 d post induction; (**C**) heatmap of commonly differentially expressed genes during preadipocyte differentiation; (**D**) validation of RNA-seq data by real-time quantitative PCR. NC, negative control.

RNA-seq was used to identify genes regulated by HOXC8 during the proliferation and differentiation of porcine preadipocytes. We first checked the knocking-down effects of *HOXC8* by siRNA in the RNA-seq data, and found that the expression of *HOXC8* was inhibited effectively in both NC-vs-Treatment groups at 0 and 3.5 d, consistent with the results obtained with qPCR in the same cells (Figure 4A). There were 450 DEGs (FC ≥ 1.5, *p* < 0.05), including 199 upregulated and 251 downregulated, and 226 DEGs, including 75 upregulated and 151 downregulated, in cells transfected with siRNA against *HOXC8* compared with those transfected with the NC sequence at 0 and 3.5 d postinduction, respectively (Tables S2-1 and S2-2).

A total of 11 common DEGs were identified, including Nuclear receptor subfamily 4 group A member (NR4A) 3, Matrix metalloproteinase 3 (MMP3), Endothelin-converting enzyme-like 1 gene, and Serpin family B member 10 (SERPINB10), between NC-vs-Treatment groups at 0 and 3.5 d postinduction (Figure 4B,C, Table S2-3). Five of these eleven DEGs had the same alteration in response to *HOXC8* knockdown in the both groups; the other six DEGs were differentially regulated by *HOXC8* at two time points, among which *SERPINB10* was a notable example: it was upregulated with a Log2(FC) = 2.91 at 0 d, and downregulated with a Log2(FC) = −1.96 at 3.5 d postinduction. A total of seven DEGs, four from 0 d and three from 3.5 d postinduction, were selected randomly for validation using qPCR, and consistent results were obtained (Figure 4D), indicating that the RNA-seq data were reliable.

**Figure 5:**
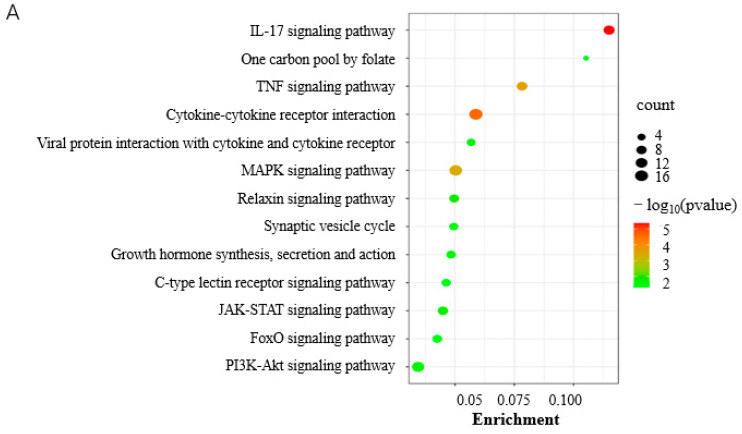

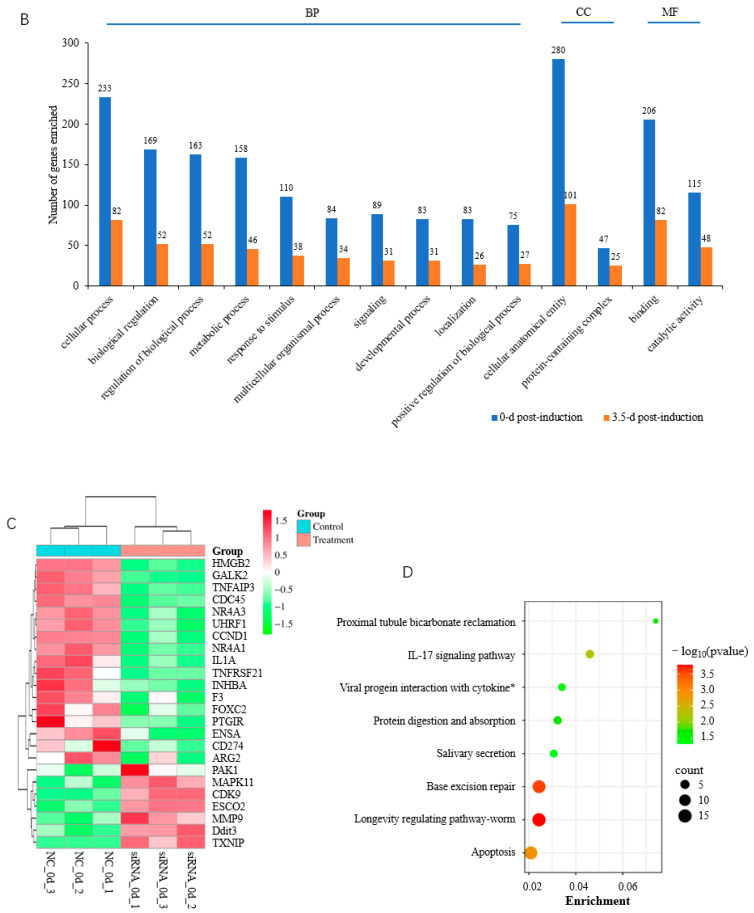
Functional analysis of DEGs. (**A**) KEGG pathways significantly enriched by DEGs at 0 d postinduction; (**B**) top GO terms enriched by DEGs at 0 and 3.5 d postinduction; (**C**) heatmap of cell cycle and proliferation genes; (**D**) KEGG pathways significantly enriched by DEGs at 3.5 d postinduction. *, viral protein interaction with cytokine and cytokine receptor.

KEGG analysis showed that DEGs were significantly involved in the IL-17 signaling pathway, TNF signaling pathway, and MAPK signaling pathway (*p* < 0.05) at 0 d postinduction (Figure 5A, Table S3-1). The DEGs were involved in various GO terms of all three functional categories, including the biological pathway (BP), cellular component (CC), and molecular function (MF) (Figure 5B). In the BP category, cellular process, biological regulation, and the regulation of biological process were among the most highly enriched GO terms. Cellular anatomical entity and binding were the most enriched in the CC and MF categories, respectively (Figure 5B).

The cells were in the stage of proliferation until being induced to differentiate and were factually not subjected to induction in the NC-vs-Treatment groups at 0 d postinduction; the RNA-seq data can reflect the effects of *HOXC8* on proliferation. DEGs related to cell proliferation were thereafter screened according to the GO term annotation and a total of 24 DEGs, 7 upregulated and 17 downregulated, were identified (Figure 5C, Table S3-2) including cell division cycle (CDC) 45, CCND1, CD274 molecule, and Cyclin-dependent kinase (CDK) 9. Among these DEGs related to cell proliferation, Establishment of sister chromatid cohesion N-acetyltransferase 2 (ESCO2) gene was the most upregulated, with a Log2(FC) = 3.0, and *CD274* was the most downregulated, with a Log2(FC) = −1.69, in response to HOXC8 knockdown.

At 3.5 d postinduction, the midterm differentiation, DEGs were significantly involved in eight pathways (*p* < 0.05) including apoptosis, the IL-17 signaling pathway, and protein digestion and absorption (Figure 5D, Table S3-3). The IL-17 signaling pathway and viral protein interaction with cytokine and cytokine receptor were the common pathways enriched significantly at both 0 and 3.5 d postinduction. Apoptosis was among the top three most significantly enriched pathways, indicating an important role in adipogenesis. It is interesting that the top GO terms enriched by DEGs at 3.5 d were identical to those at 0 d postinduction (Figure 5B).

### 3.4. Genes Involved in the Differentiation of Porcine Preadipocytes

**Figure 6:**
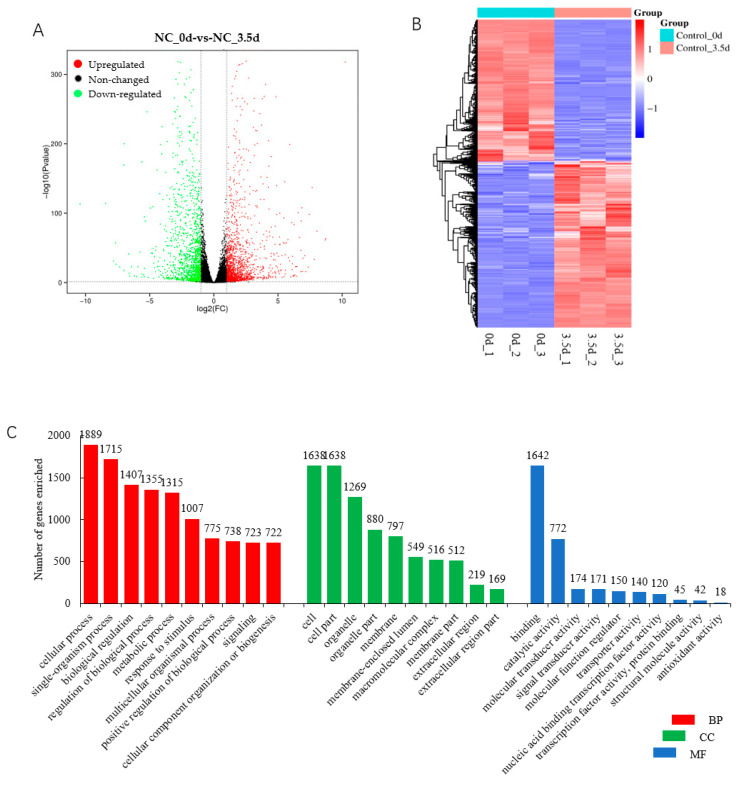

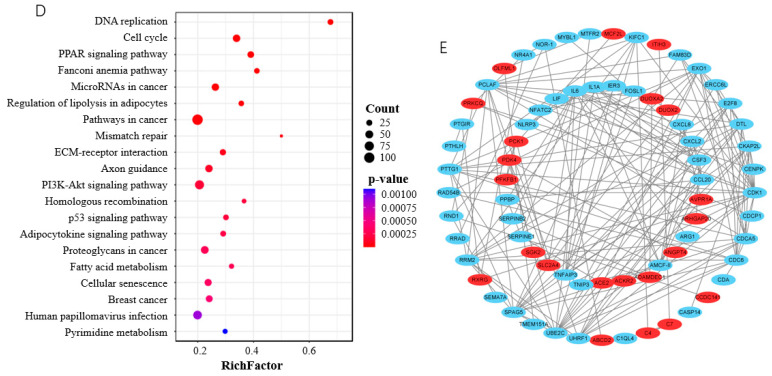
DEGs involved in differentiation of porcine preadipocytes. (**A**) Volcan plot of DEGs; (**B**) heatmap of DEGs; (**C**) top 10 GO terms in each category enriched by DEGs; (**D**) top 20 KEGG pathways significantly enriched by DEGs. BP, biological regulation; CC, cellular process; MF, molecular function; (**E**) protein–protein interaction analysis. Red indicates the genes are upregulated; blue indicates the genes are downregulated.

The total of 3008 DEGs included 1687 upregulated and 1321 downregulated in cells transfected with the NC siRNA sequence at 3.5 d postinduction compared with that at 0 d (Figure 6A,B, Table S4-1), indicating a functional role of them during the differentiation of porcine preadipocytes. In the BP category, the cellular process, biological regulation, and the metabolic process were among the top highly enriched terms, with more than 1000 genes (Figure 6C, Table S4-2). KEGG analysis showed that various pathways were significantly enriched by DEGs (*p* < 0.05), among which DNA replication and the cell cycle were the top two. Various pathways playing an important role in adipogenesis, such as the PPAR signaling pathway, the regulation of lipolysis in adipocytes, the PI3K-Akt signaling pathway, the p53 signaling pathway, the Adipocytokine signaling pathway, and fatty acid metabolism, were among the most significantly enriched pathways (*p* < 0.05) (Figure 6D, Table S4-3). PPI analysis was used to reveal the interaction between the top 100 upregulated and top 100 downregulated DEGs, and found that CDK1, IL6, Ubiquitin-conjugating enzyme E2 C (UBE2C), sperm-associated antigen 5 (SPAG5), CDC6, CDC-associated 5 (CDCA5), and PCNA-clamp-associated factor (PCLAF) had the most degrees, with a minimum ≥ 13 (Figure 6E).

## 4. Discussion

The HOX family of TFs is essential for establishing morphological diversity and controlling cell fate in a developing embryo [30,31]. Major efforts have been directed towards revealing HOX-dependent patterning since their discovery. Recently, the family was found to be frequently involved in cell proliferation and differentiation [7,8]. However, studies focused on the role of HOXC8 in fat accumulation are limited. Here, we showed that HOXC8 regulates preadipocyte proliferation and differentiation, during which several downstream genes were identified using primary cultured porcine cells. To the best of our knowledge, this is the first report on the role of porcine HOXC8 in fat formation. The results will facilitate understanding the mechanisms underlying fat accumulation regulated by HOXC8 in mammals.

Gene expression is the basis for function. Several studies have analyzed the expression of HOX family members in fat tissues, with the aim of discovering a clue to their role in adipogenesis. It was found that most of the family members were active, in vivo, in fat tissues including WAT and BAT in humans. The expression of HOX genes is tightly regulated in adipose tissues and, thus, suggests a role in adipogenesis: they not only display a depot-specific expression in WAT, but some members appear to discriminate between WAT and BAT [32]; they are differentially expressed in preadipocytes, differentiated and mature adipocytes in mice [9,33]. Here, through rechecking our previous RNA-seq data, the differential expression of HOX genes was also found in muscle tissues discriminated by fat contents, further indicating the involvement of the HOX family in fat accumulation. Among the family members identified using RNA-seq in porcine muscles, *HOXC8* was the top DEG and the level was consistently upregulated in muscles, with higher intramuscular fat contents in all three paired samples, indicating its importance as a differential phenotype between muscles.

As expected, we showed that HOXC8 is a regulator of fat accumulation through gain- and loss-of-function assays: it positively regulates the proliferation and differentiation of porcine subcutaneous preadipocytes. HOX molecules are involved in cell proliferation and differentiation, especially in tumor cells [34,35,36]. Only a few studies have focused on adipocytes, but they have revealed the diversified roles of them in fat accumulation. HOXA5 suppresses adipocyte proliferation via the transcriptional regulation of CCNE1 and inhibiting the JAK2/STAT3 signaling pathway [37]. The epigenetic dysregulation of the HOXA5 gene is associated with adipocyte hypertrophy in human obesity [38] and contributes to adipose differentiation in mice [39,40]. Consistent results for HOXA5 in adipogenesis were obtained in goats [41]. HOXC10 negatively regulates lipid droplet accumulation by suppressing the expression of lipoprotein lipase in sheep bone marrow mesenchymal stem cells [13]. HOXB7 displays a negative role in adipogenesis because it functions as a target to mediate the promoting effects of miR-24 on the adipogenesis of rat adipose-derived mesenchymal stem cells [12].

Studies in ovine HOXC8 revealed that it promotes the adipogenic differentiation of stromal vascular fractions (SVFs) [42], consistent with the promoting effects on the differentiation of porcine preadipocytes identified here, indicating a stable role of HOXC8 in adipogenesis between mammals. No reports were found on the role of HOXC8 in the proliferation of preadipocytes. Studies on chondrocytes revealed a promoting effect of HOXC8 on cell proliferation as interference with HOXC8 reduced cell proliferation [43]. Additionally, the overexpression of HOXC8 increases cell proliferation in C3H10T1/2 mouse embryonic fibroblast cells [44]. These are consistent with our results obtained in porcine preadipocytes.

Real-time PCR and RNA-seq at 0 d postinduction showed consistent results in the alteration of *CCND1* and *CCNE* transcripts in response to HOXC8 treatment. However, inconsistent results were obtained for *PCNA* with the two methods: it was not among the DEGs identified using RNA-seq, although was revealed to significantly different (*p* < 0.05) using qPCR. *PCNA* has been demonstrated as a direct downstream target gene of HOXC8 in mice, and ectopic HOXC8 enhanced its expression [44]. The deviation might be caused by the fact that the threshold for RNA-seq is higher than the change in *PCNA*.

NR4A1~3 are TFs that are highly conserved and evolutionarily ancient. They are involved in the regulation of key cellular processes such as proliferation, differentiation, and fatty acid metabolism [45,46]. Herein, the knockdown of *HOXC8* reduced the expression of NR4A1 and NR4A3 at 0 d postinduction, indicating a role of them in preadipocyte proliferation regulated by HOXC8. *NR4A3* was one of the 11 common DEGs identified in both groups at 0 and 3.5 d postinduction. It is interesting that, at 3.5 d postinduction, *NR4A3* showed an alteration contrary to that at 0 d postinduction in response to *HOXC8* knockdown. Additionally, mouse NR4A3 has been shown to inhibit adipogenesis in the 3T3-L1 cell line [47], while the downregulation of NR4A3 here suggests a positive role in porcine preadipocyte differentiation. These indicate that the regulatory network and role of NR4A3 are complicated and that different pathways and/or factors might be involved in the regulation of NR4A3 by HOXC8 during the proliferation and differentiation of porcine preadipocytes. Altogether, this is the first report highlighting the regulation relationship between HOXC8 and NR4A to the best of our knowledge.

Both DEGs involved in preadipocyte proliferation and differentiation, respectively, were enriched significantly in the IL-17 signaling pathway, indicating it is important for fat formation. An antiadipogenic role was revealed for IL-17. IL-17 suppresses adipocyte differentiation in vivo and in vitro, and moderates adipose tissue accumulation; therefore, it contributes to inhibiting obesity in mice [48,49]. IL-17A promotes the transdifferentiation of C2C12, mouse myoblast cells, into adipocytes via increasing the expression of PPARγ [50]. As for preadipocyte proliferation, few reports were found on IL-17. The results here provide a clue for the relationship between the IL-17 signaling pathway and preadipocyte proliferation.

Through analyzing data in cells transfected with siRNA NC at 0 and 3.5 d postinduction, genes involved in porcine preadipocyte differentiation were identified. The DEGs were enriched significantly (*p* < 0.05) in several pathways well known for fat accumulation, including the PPAR signaling pathway, the regulation of lipolysis in adipocytes, and fatty acid metabolism, indicating that there were no effects of siRNA NC on preadipocyte differentiation, and the data obtained from the NC-transfected cells at different induction times can be used to uncover the mechanisms underlying porcine adipogenesis. It is interesting that all the DEGs with a degree ≥ 13 in the PPI network, including *CDK1*, *IL6*, *UBE2C*, *SPAG5*, *CDC6*, *CDCA5*, and *PCLAF*, are involved in cell proliferation. CDK1, CDC6, CDCA5, and PCLAF function in cell proliferation and cell cycle regulation directly or through binding to the regulator. Numerous studies have revealed the involvement of UBE2C, IL6, and SPAG5 in cell proliferation [51,52,53]. These genes were all downregulated at 3.5 d postinduction compared with those at 0 d, indicating that the inhibition of proliferation might be one of the main processes during adipogenesis. Additionally, DNA replication and the cell cycle, involved in cell proliferation, were the top two pathways significantly enriched during preadipocyte differentiation, further suggesting the importance of inhibiting proliferation for preadipocyte differentiation.

## 5. Conclusions

Here, we showed that porcine HOXC8 promoted the proliferation and differentiation of porcine preadipocytes, and various genes involved in the regulation of HOXC8 on fat formation were identified, including 24 known proliferation-related genes. The IL-17 signaling pathway was characterized as essential for fat accumulation regulated by HOXC8. Additionally, it was revealed that the inhibition of proliferation might be one of the main processes during porcine adipogenesis. The results will contribute to further revealing the mechanisms underlying the regulation of HOXC8 on fat accumulation, and to controlling fat distribution in the future.

## Data Availability

All the relevant data are provided along with the manuscript as Supplementary Files.

## Supplementary Materials

The following supporting information can be downloaded at: https://www.mdpi.com/article/10.3390/ani13162615/s1: Table S1: Information for siRNA and primers; Table S2: Common DEGs at both 0 and 3.5 d postinduction; Table S3: Functional analysis of DEGs at 0 d postinduction; Table S4: DEGs identified between control groups at 0 and 3.5 d postinduction.
