# Supplementary material for: Effects of HOXC8 on the Proliferation and Differentiation of Porcine Preadipocytes
Source: Animals (Basel). 2023 Aug 14;13(16):2615. doi: 10.3390/ani13162615 (PMC10451666; doi:10.3390/ani13162615)
Supplement: Supplementary file 1 [file animals-13-02615-s001.zip › Table S1.docx]

Table S1-1 sequences of siRNAs against porcine HOXC8

| Names | Sequence |
| --- | --- |
| siRNA-1824 | AGAAAGAAGAGGAGGAAAATT  UUUUCCUCCUCUUCUUUCUTT |
| siRNA-2749 | AGGAAAACAACAAGGAUAATT  UUAUCCUUGUUGUUUUCCUTT |
| siRNA-3386 | CCUCCAAAUUCUAUGGCUATT  UAGCCAUAGAAUUUGGAGGTT |
| Negative control | UUCUCCGAACGUGUCACGUTT  ACGUGACACGUUCGGAGAATT |

Table S1-2 primers used for real-time quantitative PCR

| Genes | Sequence (5’-3’) |
| --- | --- |
| IBA57 | F: AATGAGTTGCCGCTTCCG  R: GGCTCCACCGTGACTTTCC |
| FKBP9 | F: CTCAGAGAGATGTGCGTTGG  R: GAGCGAGTTTCCCTTTGC |
| DDIT3 | F: CTGAAAGCAGAGCCTAATCC  R: GGCAGGGTCAAGAGTGGTG |
| C1orf52 | F: CCTTTCTCTACAACCCGCTCAAC  R: TTAGCACTCGGTGGGGCATC |
| FOSL1 | F: AGGAGACCGACAAACTGGAGG  R: GGAAAGGGAGATACAAGGCATAG |
| S100A9 | F: TGGTGATAGGAGGTGTCGG  R: GGTCTTCCAGGATGTGGTTTA |
| CA4 | F: TCGGTGATGGTGTTGCTG  R: TGCGATGTCGTCCTTGTG |
| HOXC8 | F: ATCCCGACTGTAAATCCTCTG  R: GTAAGTTTGCCTTCCGCTG |
| β-actin | F: CATCACCATCGGCAACGA  R: GCGTAGAGGTCCTTCCTGATGT |
| CCND1 | F: TGCATCTACACCGACAACTCCA  R: GTTGGAAATGAACTTCACGTCTGT |
| PCNA | F: CAATTTGGCCATGGGCGTGA  R: GGTGTCTGCATTATCTTCTGCC |
| CCNE1 | F: TGCCTTGTATCATTTCTCTT  R: GCTTCTTACTGCTCGGTG |
| β-actin | F: TGTCACGCACGATTTCC  R: CCCATCTACGAGGGCTAT |
